# Characterization and Antimicrobial Resistance of Environmental and Clinical *Aeromonas* Species Isolated from Fresh Water Ornamental Fish and Associated Farming Environment in Sri Lanka

**DOI:** 10.3390/microorganisms9102106

**Published:** 2021-10-06

**Authors:** Pavithra M. Dhanapala, Ruwani S. Kalupahana, Anil W. Kalupahana, D.P.H. Wijesekera, Sanda A. Kottawatta, Niromi K. Jayasekera, Ayona Silva-Fletcher, S.S.S. de S. Jagoda

**Affiliations:** 1 Department of Veterinary Pathobiology, Faculty of Veterinary Medicine and Animal Science, University of Peradeniya, Peradeniya 20400, Sri Lanka; pavithradhanapala92@gmail.com (P.M.D.); anilwkalupahana@yahoo.com (A.W.K.); himsiya84uni@gmail.com (D.P.H.W.); 2 Department of Veterinary Public Health and Pharmacology, Faculty of Veterinary Medicine and Animal Science, University of Peradeniya, Peradeniya 20400, Sri Lanka; ruwanikalupahana@yahoo.com (R.S.K.); sandavphp@gmail.com (S.A.K.); 3 Department of Basic Veterinary Sciences, Faculty of Veterinary Medicine and Animal Science, University of Peradeniya, Peradeniya 20400, Sri Lanka; niromikj@yahoo.com; 4 Royal Veterinary College, University of London, London NW1 0TU, UK; ASilvaFletcher@rvc.ac.uk

**Keywords:** ornamental fish, aquaculture, *Aeromonas* spp., multi-drug resistance, integrons

## Abstract

The aims of this study were to characterize and investigate antimicrobial susceptibility and presence of integrons in 161 *Aeromonas* spp. isolated from ornamental freshwater fish farming environment, apparently healthy and diseased fish. Phylogenetic analyses of the *gyrB* gene sequences identified *Aeromonas veronii* as the most abundant species (75.8%) followed by *Aeromonas* *hydrophila* (9.3%), *Aeromonas caviae* (5%), *Aeromonas jandaei* (4.3%), *Aeromonas dhakensis* (3.7%), *Aeromonas sobria* (0.6%), *Aeromonas media* (0.6%), and *Aeromonas popoffii* (0.6%). Susceptibility to thirteen antimicrobials was determined and antimicrobial resistance frequencies were: amoxicillin (92.5%), enrofloxacin (67.1%), nalidixic acid (63.4%), erythromycin (26.1%), tetracycline (23.6%), imipenem (18%), trimethoprim-sulfamethoxazole (16.8%), and gentamicin (16.8%). Multi-drug resistance (MDR) was widespread among the isolates (51.6%, 83/161) with 51.6% (63/122) *A. veronii* isolates being MDR. In addition, 68.3% of isolates had multiple antibiotic resistance (MAR) indexes higher than 0.2, suggesting that they originated from a high-risk source of contamination where antimicrobials are often used. In all, 21.7% isolates carried class 1 integrons, with 97.1% having gene cassettes, while there were 12 isolates carrying class 2 integron gene cassettes. Our findings highlight that the aquatic environment and ornamental fish act as reservoirs of multidrug resistant *Aeromonas* spp. and underline the need for a judicious use of antimicrobials and timely surveillance of antimicrobial resistance (AMR) in aquaculture.

## 1. Introduction

Members of the genus *Aeromonas* are Gram-negative bacilli and are ubiquitous in aquatic environments [1,2]. The genus *Aeromonas* comprises 36 different species, among which mesophilic, motile *Aeromonas* spp. have long been recognized as important fish pathogens [3]. They cause a wide spectrum of opportunistic infections in fresh water and brackish water fish, which are collectively known as motile *Aeromonas* septicemia (MAS). The major clinical manifestation of this disease syndrome is hemorrhagic septicemia, characterized by fin rot, dropsy, hemorrhages, and ulcers. Mesophilic *Aeromonas* species have been linked to major disease outbreaks, leading to a high mortality in cultured fresh-water food fish and ornamental fish [4,5,6], resulting in huge economic losses around the globe over the past two decades. Among mesophilic aeromonads, *A. hydrophila*, *A. caviae*, *A. sobria,* and *A. veronii* have been associated with MAS in a number of economically important fish [5,7]. *Aeromonas* is also an emerging pathogen of humans, and may cause a wide spectrum of intestinal and extra-intestinal diseases. Gastroenteritis caused by *Aeromonas* is mainly transmitted through consumption of contaminated food or water [3]. Common extra-intestinal diseases in humans caused by aeromonads include septicemia, soft tissue and wound infections, urinary tract infections, and necrotizing fasciitis, mostly in immunocompromised individuals [3,8,9]. Major *Aeromonas* species known to cause human infections include *A. caviae*, *A. dhakensis*, *A. veronii*, and *A. hydrophila* [9].

Aeromonads have been isolated from rivers, lakes, seawater, brackish water, irrigation water, chlorinated and non-chlorinated drinking water, groundwater, surface water, and wastewater. In freshwater aquaculture environments, they have been recovered from the skin and gills of healthy fish, fish excreta, pond bottom sediment, rearing water in ponds, tanks and aquaria, and ornamental fish shipping water [4,10]. Aeromonads have been shown to form biofilms on various biotic and abiotic surfaces [11,12], which enables the persistence of these pathogens in the ponds, tanks and water distribution systems associated with fish farming environments. Therefore, fresh water aquaculture environments serve as a common niche for the maintenance of high densities of mesophilic aeromonads.

Intensively cultured fresh water ornamental fish are at high risk of acquiring opportunistic bacterial infections due to the underlying stressors caused by management practices. As a result of these practices, farmers frequently use antimicrobials both therapeutically and prophylactically to control bacterial infections. Enrofloxacin, erythromycin, amoxicillin, chloramphenicol, florfenicol, sulfonamides, oxolinic acid, oxytetracycline, and tetracycline are the commonly used antimicrobials in the aquaculture sector [13,14] and these are either incorporated into fish feed (medicated feed) or added directly in to the water (baths). Uneaten food and fish feces may retain antimicrobial residues within the aquatic environment [15]. The lack of standardized guidelines when using antimicrobials in ornamental fish aquaculture has led to frequent overuse and abuse of antimicrobials by farmers. The use of antimicrobials to treat non-bacterial infections, suboptimal use of antimicrobials, antimicrobial shot-gunning (use of many different antimicrobials one after the other), and use of the same antimicrobial over a prolonged period of time are examples of misusing antimicrobials in aquaculture. Over the counter availability and the off-label use of antimicrobials aggravate these practices [16,17,18]. The long-term misuse and abuse of antimicrobials in fresh water ornamental fish culture and subsequent passage of these antimicrobials into the aquatic environment is likely to result in the emergence of AMR in aquatic bacteria. AMR in bacteria can be acquired by selective mutations in the genome and these can be transferred to clinically important piscine and human pathogens through horizontal gene transfer.

Multiple antimicrobial resistant *Aeromonas* isolates have been reported globally associated with ornamental fish and food fish culture [1,19]. Single and multiple antimicrobial resistance has been shown to commonly used antimicrobials in aquatic sector, such as erythromycin, tetracycline, chloramphenicol, nalidixic acid, and nitrofurantoin. These studies have also shown aeromonads with high resistance to amoxycillin, tetracycline and nalidixic acid [8,17,20,21,22,23].

Widely distributed unusual bacterial genetic elements (known as integrons) [24,25] may combine with mobile genetic elements (MGE), such as plasmids and transposons, to give rise to mobile integrons that may spread antimicrobial resistance to bacteria in the environment [1,26]. Depending on the encoding integrase gene, integrons are divided into four classes. Classes 1, 2 and 3 are mobile and class 4 integrons or superintegrons are regarded as non-mobile [24,25,26,27]. Class 1 integrons are the most common type among Gram-negative bacteria [25,27] and in *Aeromonas* spp. they are the most prevalent and best documented [24].

Ornamental fish keeping has become a popular hobby among many people and more than 100 countries in the world are involved in the global ornamental fish trade [7]. The ornamental fish industry in Sri Lanka has expanded to be a large business over the past three decades. The country exports nearly 4.2% of the world’s demand for ornamental fish [28] including guppy (*Poecilia reticulata*), neon tetra (*Paracheirodon innesi*), platy (*Xiphophorus maculatus*), swordtails (*Xiphophorus helleri*), and molly (*Poecilia sphenops*). This accelerated growth in ornamental fish aquaculture has led to the intensification of farming practices creating a need to rely on chemicals to safeguard the health of fish. Considering that little is known about the role of aeromonads as a reservoir of antimicrobial resistance in the ornamental fish aquaculture environment of Sri Lanka, we evaluated the antimicrobial susceptibility profiles of *Aeromonas* spp. isolated from diseased fish, healthy fish and associated aquatic environments. Further, we also investigated the existence of integrons associated with the antimicrobial resistant *Aeromonas* spp.

## 2. Materials and Methods

### 2.1. Sample Collection

#### 2.1.1. Fish Farming Environment

Twenty-four ornamental fish breeding/rearing farms located in the central, north-western, north central and western provinces of Sri Lanka were visited during the period from July, 2020 to March, 2021 for the collection of samples. Environmental samples from each farm included a 50 mL sample of effluent water (collected to a sterile centrifuge tube), a sample of pond/tank sediment (collected into sterile 300 mL plastic bottles with corresponding pond/tank water) and a sample of biofilm removed/scraped from aquarium tubing/pipes (collected into 50 mL centrifuge tubes filled with 15 mL sterile distilled water). Environmental samples were transported to a laboratory in a cool box and processed for culturing soon after arriving at the laboratory within 24 h after collection.

#### 2.1.2. Apparently Healthy Fish

Two to five apparently healthy tropical fresh water ornamental fish (any of the fish species guppy, platy, molly, swordtail and goldfish, depending on the availability) were collected from each farm. Live fish were transported in polythene bags with oxygenated water and transferred to 10 L glass aquaria separately.

#### 2.1.3. Diseased Fish

Live, moribund fresh water ornamental fish presenting clinical signs of septicemia (hemorrhages on skin, abdominal distension, skin ulcers, fin rot) were collected from the above farms if such fish were available at the time of visiting and transported to the laboratory in polythene bags with oxygenated water. In addition, septicemic moribund fish submitted to the Centre for Aquatic Animal Disease Diagnosis and Research (CAADDR), University of Peradeniya for disease investigations were also included.

### 2.2. Isolation of Aeromonads and Phenotypic Characterization

Effluent water and pond sediment samples were centrifuged at 6000 rpm for 15 min. After removing the supernatant carefully, a loopful of sediment from each sample was plated directly onto trypticase soy agar (TSA; HiMedia, Mumbai, India) (modified from [29,30]). Biofilm samples were also centrifuged (as mentioned above) and the sediments were enriched in nutrient broth for 24 h at room temperature. After the incubation, one loopful of nutrient broth was plated onto TSA. From apparently healthy fish, skin mucous samples were collected using sterile swabs and cultured on TSA. Moribund, diseased fish were humanely euthanized using an overdose of Tricane-methanesulphonate (MS 222; Sigma-Aldrich, St. Louis, MO, USA) and the kidneys were cultured aseptically on TSA. Kidney samples were also obtained during autopsies of moribund, septicemic fish submitted to CAADDR for disease investigations and cultured on TSA. If the fish showed clinical signs of external bacterial infections such as skin ulcers and/or fin rot, swabs taken from the lesions were also cultured on TSA. All culture plates were incubated at room temperature (27–28 °C) for 24–48 h. All different colony types in each plate were subjected to Gram-staining and all Gram-negative rod-shaped isolates were subcultured on Aeromonas starch DNA agar (HiMedia, Mumbai, India) and Glutamate phenol red (GSP) agar (HiMedia, Mumbai, India). Isolates that showed luxuriant growth on Aeromonas starch DNA agar and yellow color colonies on GSP agar were subcultured on TSA for 24 h at 28 °C and were subjected to cytochrome oxidase test, catalase test, motility determination, oxidation-fermentation test and susceptibility to vibriostatic agent 0/129 (10 µg, Oxoid, Hampshire, UK). Isolates which were Gram-negative, cytochrome oxidase positive, catalase positive, motile, fermentative and resistant to vibrio static agent were phenotypically identified as presumptive aeromonads.

### 2.3. DNA Extraction, Genetic Characterization and Phylogenetic Analysis

#### 2.3.1. Genus Identification

Genomic DNA was extracted from all presumptive *Aeromonas* isolates using a commercial DNA extraction kit (ReliaPrep gDNA tissue miniprep system, Promega, Madison, WI, USA) according to the manufacturer’s instructions. The 16S rRNA gene from each isolate was amplified by PCR using *Aeromonas* genus specific 16S rRNA primers [31] (Table 1). Reaction was performed in a final volume of 50 µL containing 2.5 µL of 10× reaction buffer (TaKaRa, Shiga, Japan), 4 µL of 25 mM MgCl_2_ (Promega, Madison, WI, USA), 2 µL of 2.5 mM deoxyribonucleotide mix (TaKaRa, Shiga, Japan), 0.3 µL of Taq DNA polymerase (5 U/µL, TaKaRa, Shiga, Japan), 0.4 µL of 50 µM of each forward and reverse primer and 5 µL of DNA sample. The PCR conditions were as follows: an initial denaturation step at 93 °C for 3 min; 35 subsequent cycles of denaturation at 94 °C for 1 min, annealing at 56 °C for 1 min, and elongation at 72 °C for 2 min; and a final extension at 72 °C for 10 min. Field strains *A. hydrophila* Ae34 (draft genome accession number BAXY01000001 to BAXY01000028 [32]) and *A. veronii* Ae52 (draft genome accession number BDGY01000001-BDGY01000080 [33]) were used as positive controls in PCR experiments.

All PCRs were performed using a GeneAmp^®^ PCR System 9700 thermal cycler (Applied biosystems, Foster city, CA, USA). Amplified products were analyzed by electrophoresis on 1% agarose-TBE gels stained with ethidium bromide and visualized in a gel documentation system (Geneflash, Syngene, gel imaging, Cambridge, UK).

Then, from the isolates that were identified as belonging to the genus *Aeromonas* by 16S rRNA PCR, up to three to seven isolates were selected per farm, depending on the isolate’s origin (source) and the antimicrobial susceptibility profiles for subsequent characterization. Accordingly, a total of 101 environmental isolates (from effluent water, pond sediment and biofilm) and 42 commensal isolates (from apparently healthy fish) were included in the further analysis. In addition, 42 clinical isolates recovered from diseased fish were also included.

**Table 1:** Sequences of oligonucleotide primers used in this study.

Primer	Sequence	Reference
*16S rRNA forward*	5′ AGAGTTTGATCATGGCTCAG 3′	[31]
*16S rRNA reverse*	5′ GGTTACCTTGTTACGACTT 3′	
*gyrB 3F*	5′ TCCGGCGGTCTGCACGGCGT 3′	[34]
*gyrB 14R*	5′ TTGTCCGGGTTGTACTCGTC 3′	
*hep35*	5′ TGCGGGTYAARGATBTKGATTT 3′	[35]
*hep36*	5′ CARCACATGCGTRTARAT 3′	
*IntI1.F*	5′ GGG TCA AGG ATC TGG ATT TCG 3′	[36]
*IntI1.R*	5′ ACA TGC GTG TAA ATC ATC GTC G 3′	
*hep58*	5′ TCATGGCTTGTTATGACTGT 3′	[35]
*hep59*	5′ GTAGGGCTTATTATGCACGC 3′	
*hep74*	5′ CGGGATCCCGGACGGCATGCACGATTTGTA 3′	[37]
*hep51*	5′ GATGCCATCGCAAGTACGAG 3′	

#### 2.3.2. Species Identification

In order to identify aeromonads at species level, a fragment of approximately 1100 bp of the *gyrB* gene from each isolate was amplified by PCR using primers *GyrB3F* and *GyrB14R* (Table 1) in a GeneAmp^®^ PCR System 9700 thermal cycler (Applied biosystems, Foster city, CA, USA). Reaction was performed in a final volume of 50 µL containing 5 µL of 10× reaction buffer (TaKaRa, Shiga, Japan), 4 µL of 25 mM MgCl_2_ (Promega, Madison, WI, US), 4 µL of 2.5 mM deoxyribonucleotide mix (TaKaRa, Shiga, Japan), 0.2 µL of Taq DNA polymerase (5 U/µL, TaKaRa, Shiga, Japan), 1 µL of 10 µM of each forward and reverse primer and 1 µL of genomic DNA. The amplification program consisted of initial denaturation at 94 °C for 3 min, followed by 35 cycles of denaturation at 94 °C for 15 s, annealing at 57 °C f or 30 s and extension at 72 °C for 45 s. Final extension was achieved at 72 °C for 3 min [34]. Amplified products were analyzed by electrophoresis on 1% agarose in 1X Tris-borate-EDTA (TBE gels) stained with ethidium bromide and visualized in a UV transilluminator (Geneflash Syngene gel imaging, Cambridge, UK).

After amplification, the PCR products were resolved by electrophoresis in 1% agarose gels (in 1X TBE) stained with ethidium bromide and visualized on a gel documentation system. PCR-amplified *gyrB* products were purified and directly sequenced by Macrogen, South Korea. The DNA sequence was double-checked by sequencing both strands using primers *GyrB3F* and *GyrB14R* for forward and reverse reactions, respectively. Sequences were viewed, aligned and manually edited to resolve ambiguous positions using MEGA-X [38] and confirmed by interrogation of the GenBank DNA sequence database using BLAST algorithms (http://www.ncbi.nlm.nih.gov/BLAST/, accessed on 9 June 2021).

#### 2.3.3. Nucleotide Sequence Accession Numbers

Nucleotide sequences have been deposited in DDBJ/EMBL/GenBank databases under the accession numbers LC644207 to LC644367.

#### 2.3.4. Phylogenetic Analysis

Partial *gryB* sequences of *Aeromonas* isolates (*n* = 161) and reference strains retrieved form Genbank (*n* = 10) were aligned in MEGA-X using CLUSTAL-W. The final length of the alignment used in the phylogenetic analysis was 961 bp. A phylogenetic tree was constructed by the neighbor-joining method [39] using the MEGA-X program. Genetic distances were computed by using Tamura’s three-parameter model [40]. In order to statistically evaluate the tree, bootstrapping was carried out with data resampled 1000 times.

### 2.4. Antimicrobial Susceptibility Testing

Antimicrobial susceptibility of each *Aeromonas* isolate was determined against 13 antimicrobials on Mueller-Hinton agar by the disk diffusion method, using commercially available disks (Oxoid, Hampshire, UK) according to the guidelines of the Clinical and Laboratory Standards Institute (CLSI). The antimicrobial agents tested included amoxycillin (10 µg), nalidixic acid (30 µg), rifampicin (5 µg), chloramphenicol (30 µg), doxycycline (30 µg), erythromycin (15 µg), enrofloxacin (5 µg), tetracycline (30 µg), nitrofurantoin (300 µg), trimethoprim-sulfamethoxazole (25 µg), gentamicin (10 µg), ceftazidime (30 µg) and imipenem (10 µg). Results were interpreted as susceptible (S), intermediate (I) and resistant (R) based on the CLSI interpretive criteria for *Aeromonas* species (CLSI VET04 [41], CLSI M45 [42] and CLSI M100 [43]). (Diameters of the inhibition zones (mm) used for interpretation are as follows; Nalidixic acid, R ≤ 13, I-14-18, S ≥ 19; Doxycycline, R ≤ 10, I-11-13, S ≥ 14; Nitrofurantoin, R ≤ 14, I-15-16, S ≥ 17 (CLSI M100, inhibitory zones for *Enterobacteriacaea*); Amoxicillin, R ≤ 6, S > 7; Rifampicin, R < 8, I-7-9, S > 10; Chloramphenicol, R ≤ 12, I-13-17, S ≥ 18; Erythromycin, R ≤ 14,S > 14; Enrofloxacin, R ≤ 32, S > 32; Tetracycline, R ≤ 11, I-12-14, S ≥ 15; Trimethoprim-sulfamethoxazole, R ≤ 21, S > 21; Gentamicin, R ≤ 18, S > 18; Ceftazidime, R ≤ 17, I-18-20, S ≥ 21; Imipenem, R ≤ 19, I-20-22, S ≥ 23).

### 2.5. PCR Amplification of Integrons and Determination of the Class of Integrons

The genomic DNA of all isolates were screened by PCR for the presence of integrons using degenerative primers *hep 35* and *hep 36* which hybridize to conserved regions of integron-encoded integrase genes *intI1*, *intI2*, and *intI3* described previously [35]. PCRs were performed with the cycling conditions consisting of an initial denaturation at 93 °C for 3 min, followed by 30 cycles of amplification as denaturation at 94 °C for 30 sec, annealing at 55 °C for 30 sec and extension at 72 °C for 45 sec, and a final extension at 72 °C for 10 min. Reaction was performed in a final volume of 50 µL containing 5 µL of 10× reaction buffer (with MgCl_2_) (TaKaRa, Shiga, Japan), 0.5 µL of 2.5 mM deoxyribonucleotide mix (TaKaRa, Shiga, Japan), 0.3 µL of Taq DNA polymerase (5 U µL^−1^; TaKaRa, Shiga, Japan), 1 µL of 50 µM of each forward and reverse primer and 2 µL of DNA.

The PCR products were subjected to electrophoresis on 1% agarose gels in 1X TBE buffer, stained with ethidium bromide and visualized in a gel documentation system (Geneflash Syngene gel imaging, Cambridge, UK).

All isolates that successfully amplified a 491 bp fragment in integrase PCR were considered integron positive. The class of integron was determined by analyzing integrase PCR products by restriction fragment length polymorphism (RFLP) following digestion using either *Rsa*I or *Hin*fI restriction enzymes. After digesting PCR products at 37 °C for 16 h, resulting DNA fragments were analyzed in 2% high resolution agarose (in 1X TBE) stained with ethidium bromide. Integron classification was done according to the fragment pattern described previously [37].

Isolates carrying class 1 integrons were further confirmed by another PCR [36]. Reaction was performed in a final volume of 50 µL containing 5 µL of 10× reaction buffer (TaKaRa, Shiga, Japan), 2 µL of 25 mM MgCl_2_ (Promega, Madison, WI, US), 2 µL of 2.5 mM deoxyribonucleotide mix (TaKaRa, Shiga, Japan), 0.2 µL of Taq DNA polymerase (5 U/µL, TaKaRa, Shiga, Japan), 1 µL of 50 µM of each forward and reverse primer and 5 µL of genomic DNA. The amplification program consisted of initial denaturation at 94 °C for 5min, followed by 30 cycles of denaturation at 94 °C for 30 s, annealing at 62 °C for 30 s and extension at 72 °C for 60 s. Final extension was achieved at 72 °C for 8 min [34]. Amplified products were analyzed by electrophoresis on 1% agarose in 1× Tris-borate-EDTA (TBE gels) stained with ethidium bromide and visualized in a UV transilluminator (Geneflash Syngene gel imaging, Cambridge, UK).

### 2.6. Amplification of the Gene Cassettes Regions of Class 1 and Class 2 Integrons

Class 1 integron cassette regions were amplified in the isolates carrying class 1 integrons using *hep 58* and *hep 59* as described previously [35]. PCR amplification was carried out in a final volume of 50 µL containing 5 µL of 10× reaction buffer (with MgCl_2_) (TaKaRa, Shiga, Japan), 1 µL of 2.5 mM deoxyribonucleotide mix (TaKaRa, Shiga, Japan), 0.5 µL of Taq DNA polymerase (5 U/µL, TaKaRa, Shiga, Japan), 1 µL of 50 µM of each forward and reverse primer and 2 µL of DNA. PCRs were performed with the cycling conditions consisted of an initial denaturation at 93 °C for 3 min, which is followed by 30 cycles of amplification as denaturation at 94 °C for 30 s, annealing at 55 °C for 30 s and extension at 72 °C for 4 min. The final extension was carried out at 72 °C for 10 min.

Class 2 integron cassette regions were amplified by PCR using *hep 74* and *hep 51* according to a protocol described previously [44]. PCR amplification was carried out in a final volume of 50 µL containing 5 µL of 10× reaction buffer (with MgCl_2_) (TaKaRa, Shiga, Japan), 0.5 µL of 2.5 mM deoxyribonucleotide mix (TaKaRa, Shiga, Japan), 0.4 µL of Taq DNA polymerase (5 U/µL, TaKaRa, Shiga, Japan), 1.5 µL of 50 µM of each forward and reverse primer and 2 µL of DNA. PCRs were performed with the cycling conditions consisted of an initial denaturation at 94 °C for 3 min, which is followed by 35 cycles of amplification as denaturation at 94 °C for 30 s, annealing at 55 °C for 1.5 min and extension at 72 °C for 3 min. The final extension was carried out at 72 °C for 7 min.

### 2.7. Statistical Analysis

Logistic regression models were performed to determine the degree of association between the dependent variable (presence of multi drug resistance/presence of an integron/presence of a class 1 integron in environmental and commensal isolates of *Aeromonas* species), and following independent variables; scale of the farm, source of sampling, bacterial species. Analyses were performed using R statistical software 4.1.1 (R Development Core Team 2008).

## 3. Results

Twenty-four ornamental fish breeding farms were visited, including 19 (79.2%) polyculture farms and 5 (20.8%) monoculture farms, and samples were taken. Out of 5 monoculture farms, 3 (60%) had guppy (*Poecilia reticulata*) while 1 (20%) each had swordtail fish (*Xiphophorus helleri*) and platy (*Xiphophorus maculatus*). Half of the visited farms (50%., *n* = 12) supplied their fish only to the local market while rest of the farms supplied fish to both export and local markets. Based on their production capacity, farms could be categorized into 8 small scale (monthly production < 3000 fish), 10 medium scale (monthly production 3000–10,000 fish), and 6 large scale (monthly production > 10,000 fish).

### 3.1. Identification of Aeromonas *spp.*

#### 3.1.1. Phylogenetic Analysis

A total of 246 Gram-negative, oxidase positive bacterial isolates were recovered upon culturing 96 environmental and healthy fish samples collected from 24 ornamental fish breeding farms, of which 178 were phenotypically identified as presumptive *Aeromonas* species.

Together with forty-six clinical isolates of phenotypically identified aeromonads recovered from 29 septicemic fish, a total of 224 presumptive *Aeromonas* isolates were subjected to *Aeromonas* genus specific 16S rRNA PCR. From 16S rRNA PCR positive isolates (*n* = 213), 185 independent (non-redundant) isolates (143 environmental and 42 clinical) were selected based on the sample location, sample type and antimicrobial resistance phenotype. Of those isolates, 161 (87%) were identified as *Aeromonas* species (Table S1) and the others were identified as *Citrobacter* species (*n* = 6, 3.2%), *Serratia* species (*n =* 5, 2.7%), *Klebsiella pneumoniae* (*n* = 3, 1.6%), *Enterobacter* species (*n* = 3, 1.6%), *Morganella morganii* (*n* = 2, 1.08%), *Commamonas aquatica* (*n* = 1, 0.5%), *Plesiomonas shigelloides* (*n* = 1, 0.5%), *Shewanella decolorationis* (*n* = 1, 0.5%), *Phytobacter diazotrophicus* (*n* = 1, 0.5%) and *Pseudaeromonas sharmana* (previously known as *Aeromonas sharmana*) (*n* = 1, 0.5%) by sequencing of the *gyrB* gene.

According to the *gyrB* gene sequence analysis, *Aeromonas* isolates (*n* = 161) were identified as belonging to eight different species of *Aeromonas*: *A. veronii* (*n* = 122, 75.8%), *A. hydrophila* (*n* = 15, 9.3%), *A. caviae* (*n* = 8, 5%), *A. jandaei* (*n* = 7, 4.3%), *A. dhakensis* (*n* = 6, 3.7%), *A. sobria* (*n* = 1, 0.6%), *A. media* (*n* = 1, 0.6%) and *A. popoffii* (*n* = 1, 0.6%)(Table S1). Sequence similarity between *Aeromonas* strains ranged from 97.25% to 100%. Intraspecies similarity for aeromonad isolates was above 98% for *A. veronii*, *A. hydrophila*, *A. dhakensis*, *A. sobria* and *A. popoffii*; 97.70–99.31% for *A. caviae*; 97.53–99.01% for *A. jandaei* and 97.25% for *A. media.*

**Figure 1:**
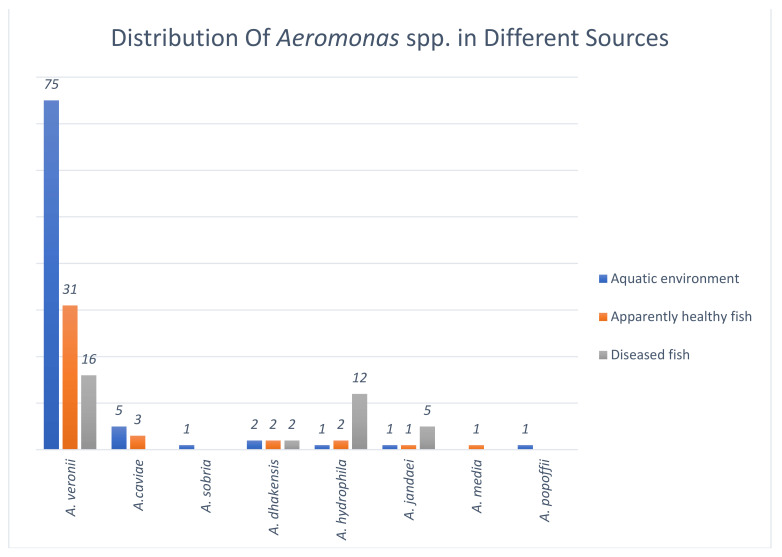
Distribution of *Aeromonas* species in the aquatic environment, apparently healthy and diseased ornamental fish.

Table 2 shows the distribution of these 161 *Aeromonas* isolates based on different sources of isolation. This included 26 isolates from effluent water, 40 isolates from pond sediment, 20 isolates from biofilms, 40 commensal isolates and 35 clinical isolates. In the present study, *A. veronii* was the most prevalent species in all sampling sources i.e., the aquatic environment, apparently healthy ornamental fish and diseased fish (Figure 1).

**Figure 2:**
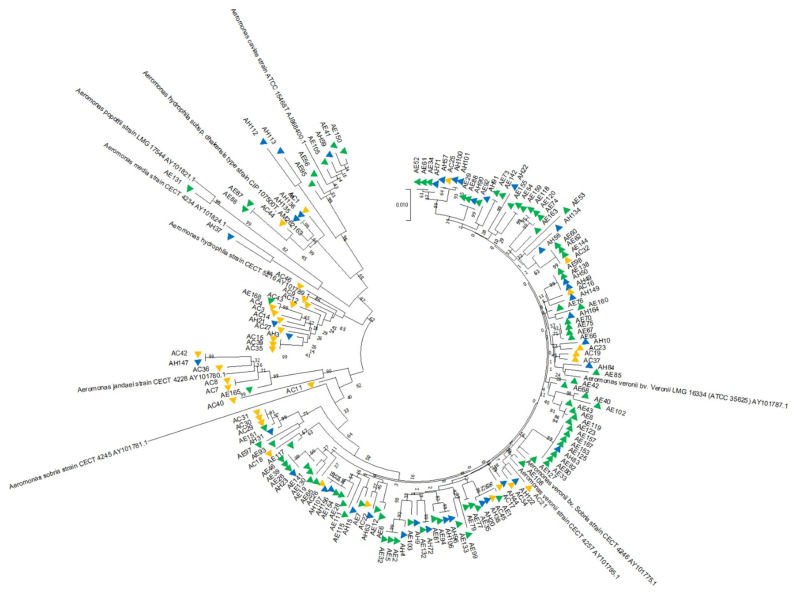
Phylogenetic tree of *Aeromonas* spp. based on *gyrB* sequences using neighbor-joining method with bootstrap replication with 1000. AE: Aeromonads isolated from environment; AH: aeromonads isolated from healthy fish; AC: aeromonads isolated from diseased ornamental fish. The color of triangle next to the isolate name represents the source of isolation; green—environmental, blue—healthy ornamental fish, yellow—diseased ornamental fish.

The neighbor-joining phylogenetic tree constructed by using partial *gyrB* gene sequences showed distinct clustering of species with high bootstrap values, ranging from 99% to 100% (Figure 2 and Figure S1). The GenBank accession numbers of partial *gyrB* gene sequence of reference strains used in the phylogenetic analysis were AY101795, AY101775, AY101787, AJ868400, AY101781, AM262163, AY101789, AY101780, AY101824, and AY101821.

#### 3.1.2. Species Distribution of *Aeromonas* among Isolates from the Aquatic Environment

*Aeromonas* isolates recovered from the ornamental fish farming environment (*n* = 86) represented seven distinct species. The most abundant species, was *A. veronii* (*n* = 75, 87.2%) followed by *A. caviae* (*n* = 5, 5.8%), *A. dhakensis* (*n* = 2, 2.3%), *A*. *hydrophila* (*n* = 1, 1.2%), *A. sobria* (*n* = 1, 1.2%), *A. jandaei* (*n* = 1, 1.2%) and *A. popoffii* (*n* = 1, 1.2%). *A. veronii* was the most abundant species in all three environmental sample types and represented 88.5% (23/26) of effluent water isolates, 90% (36/40) of the pond sediment isolates and 80% (16/20) of the biofilm isolates. It is noteworthy to mention the very low prevalence of *A. hydrophila* among the environmental aeromonads isolated from these ornamental fish farms.

#### 3.1.3. Species Distribution of *Aeromonas* among Isolates from Apparently Healthy Ornamental Fish

Commensal aeromonads isolated from apparently healthy fish (*n* = 40) were identified as belonged to six different species. *A. veronii* (*n* = 31, 77.5%) was the most common species, followed by *A. caviae* (*n* = 3, 7.5%), *A. dhakensis* (*n* = 2, 5%), *A*. *hydrophila* (*n* = 2, 5%), *A. jandaei* (*n* = 1, 2.5%) and *A. media* (*n* = 1, 2.5%). *A. hydrophila* and *A. dhakensis* were isolated only from guppy but not from other fish species used in this study.

#### 3.1.4. Species Distribution of Clinical *Aeromonas* Isolates from Ornamental Fish

Clinical *Aeromonas* isolates (*n* = 35) from diseased ornamental fish belonged to four different species of which the most abundant was *A*. *veronii* (*n* = 16, 45.7%), which was followed by *A. hydrophila* (*n* = 12, 34.3%), *A. jandaei* (*n =* 5, 14.3%) and *A. dhakensis* (*n* = 2, 5.7%). It is noteworthy to mention the high prevalence of *A. hydrophiila* and *A. jandaei* among clinical isolates compared to that of environmental and commensal isolates.

### 3.2. Antimicrobial Susceptibility

A total of 161 *Aeromonas* spp. were subjected to antimicrobial susceptibility testing, including 86 of environmental, 40 of commensal and 35 of clinical origin, respectively.

**Figure 3:**
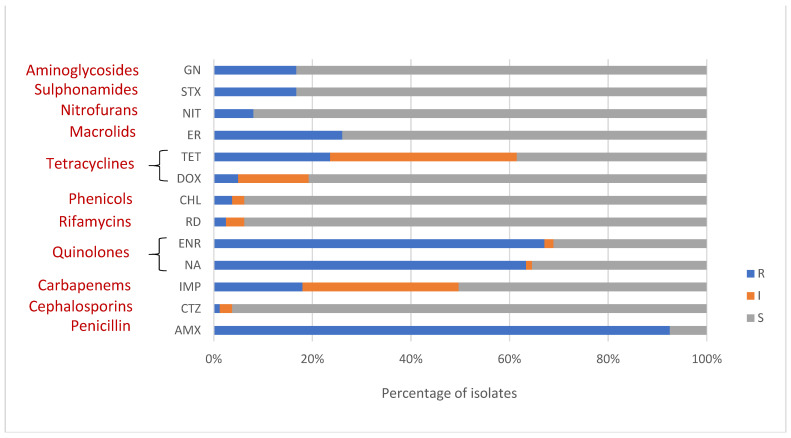
Antimicrobial susceptibility patterns of 161 *Aeromonas* isolates for antimicrobials tested and their classes (in dark red); amoxicillin (AMX), nalidixic acid (NA), enrofloxacin (ENR), rifampicin (RD), chloramphenicol (CHL), doxycycline (DOX), tetracycline (TET), erythromycin (ER), nitrofurantoin (NIT), trimethoprim-sulfamethoxazole (STX), gentamicin (GN), ceftazidime (CTZ) and imipenem (IMP) are shown. (R—resistant, I—intermediate, S—susceptible).

In this study, 88.82% (143/161) showed resistance to more than one antimicrobial. Only one isolate (0.62%) showed sensitivity to all antimicrobials tested. A majority of isolates was resistant to amoxycillin (92.5%, *n* = 149) which could be attributed to the intrinsic resistance of aeromonads against penicillins. Alarmingly, many *Aeromonas* isolates exhibited resistance to enrofloxacin (67.1%, *n* = 108) and nalidixic acid (63.4%, *n* = 102). Isolates also presented a considerable resistance rate to erythromycin (26.1%, *n* = 42), tetracycline (23.6%, *n* = 38), imipenem (18%, *n* = 29), gentamicin (16.8%, *n* = 27) and trimethoprim-sulfamethoxazole (16.8%, *n* = 27). Meanwhile, resistance to nitrofurantoin (8.1%, *n* = 13), doxycycline (5%, *n* = 8), chloramphenicol (3.7%, *n* = 6), rifampicin (2.5%, *n* = 4), and ceftazidime (1.2%, *n* = 2) was also found but in low proportion of isolates (Figure 3).

**Figure 4:**
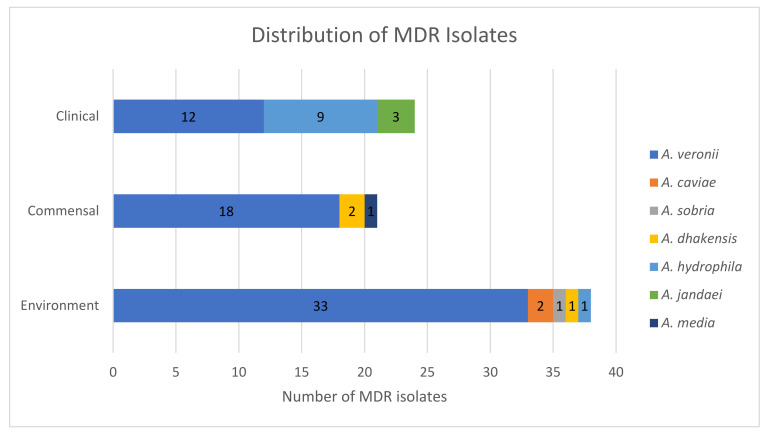
Distribution of MDR isolates among environmental, commensal and clinical aeromonads.

Out of 161 isolates, 83 (51.6%) were MDR (Figure 4). Of those, 18 isolates (11.2%) showed resistance to more than five antimicrobials, including one *A. dhakensis* isolate, which showed resistance to eleven antimicrobials and sensitivity only to imipenem. *A. veronii* represented 75.9% (63/83) of MDR isolates. On the other hand, nearly half of *A. veronii* isolates (51.6%, 63/122) were MDR. High level of resistance was observed in *A. veronii*, which was the most abundant species, against amoxicillin (91%, *n* = 111), nalidixic acid (65.6%, *n* = 80) and enrofloxacin (63.9%, *n* = 78) which belong to beta-lactams, synthetic quinolones and fluoroquinolones, respectively. Further, a considerable number of *A. veronii* isolates showed resistance or intermediate resistance to tetracyclines (65.6%, *n* = 80) and imipenem (54.1%, *n* = 66). Other MDR isolates belonged to *A. hydrophila, A. dhakensis, A. jandaei, A. caviae, A. media* and *A. sobria.* According to the statistical analysis, multi drug resistance in environmental and commensal *Aeromonas* isolates is significantly lower (*p* < 0.05) in small scale farms compared to that of the reference group (large scale farms).

The distributions of antimicrobial susceptibility of different species of *Aeromonas* isolated in the present study against thirteen antimicrobials tested are presented in Table 3.

In this study, 68.3% (*n* = 110) isolates showed MAR index higher than 0.2, of which 78.1% (*n* = 86) were *A. veronii* isolates. The highest MAR index of 0.85 was observed in an isolate of *A. dhakensis* from the skin mucus of an apparently healthy guppy which was found to be resistant to 11 out of 13 tested antimicrobials. The second highest MAR index of 0.54 was observed in six different isolates that comprised of 4 isolates of *A. veronii*, and 1 isolate each *A. hydrophila* and *A. dhakensis.*

**Figure 5:**
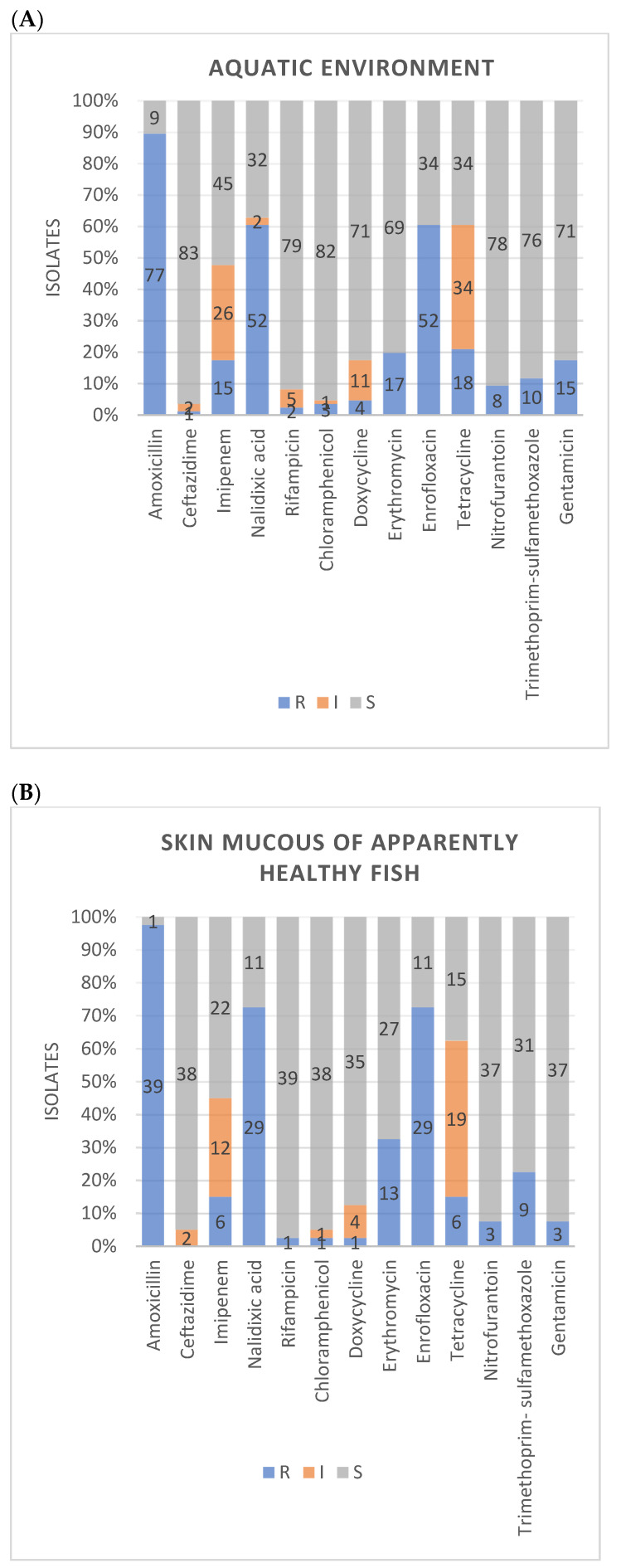

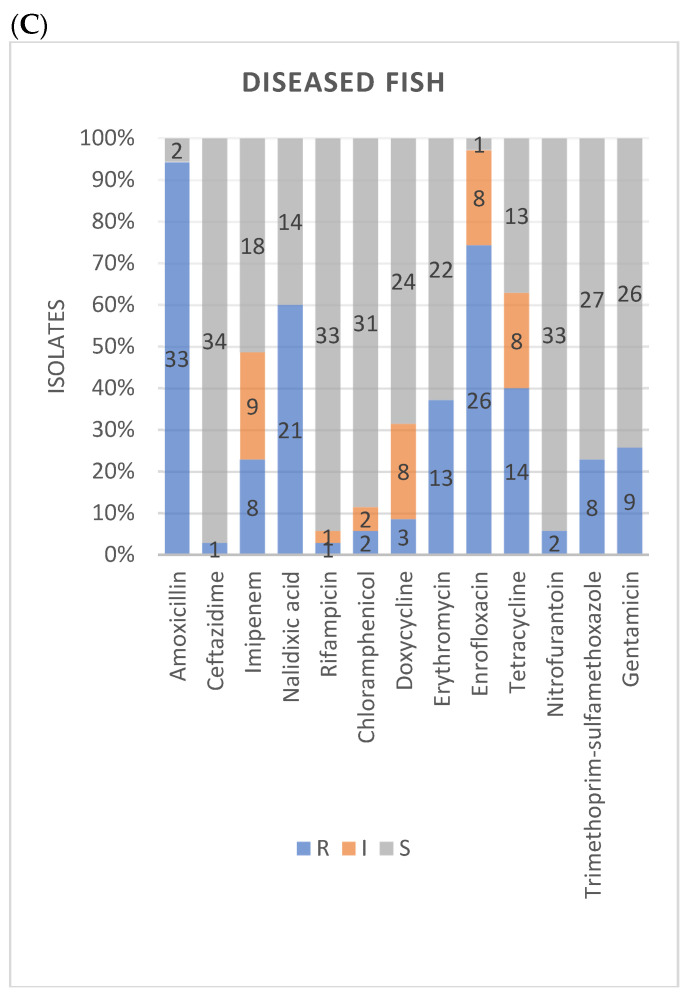
Antimicrobial susceptibility pattern of *Aeromonas* spp. isolated from different sample types; (**A**) Aquatic environment; (**B**) skin mucous of apparently healthy fish; (**C**) diseased fish (R—resistant, I—intermediate, S—susceptible).

Aeromonads from environment, apparently healthy fish and diseased fish showed more or less similar resistance levels for many antimicrobials tested (Figure 5A–C). Exceptions were comparatively higher level of resistance observed in clinical aeromonads against tetracycline than commensal and environmental isolates, and high level of resistance in fish isolates (both clinical and commensal) against trimethoprim-sulfamethoxazole compared to environmental isolates.

### 3.3. Detection of Class 1 and Class 2 Integrons

Using PCR primers *hep 35* and *hep 36* targeting conserved regions of integron-encoded integrase genes *intI1*, *intI2*, and *intI3* integrons were detected in 32 of the 161 Aeromonas isolates included in the study (19.9%). They represented 34.4% isolates of environmental origin (*n* = 11), 37.5% isolates from apparently heathy fish (*n* = 12) and 28.1% isolates from diseased fish (*n* = 9) isolates respectively.

Analysis of integrase PCR products by restriction fragment length polymorphism (RFLP), allowed identification of 31 class 1 integron bearing isolates and 1 class 2 integron bearing isolate. In order to capture the class 1 integron bearing isolates more accurately, a PCR targeting class 1 integron-related integrase (intI1) gene was occupied and after comparing RFLP results and IntI1 gene amplification results, a total of 35 (21.7%, 35/161) aeromonads were identified as carrying class 1 integrons. These comprised of 15 environmental isolates (9.3%,15/161), 12 isolates from apparently healthy fish (7.5%, 12/161) and 8 clinical isolates (5%, 8/161). Class 1 integrons were detected in 24 *A. veronii*, 4 *A. jandaei*, 2 *A. hydrophila*, 2 *A*. *dhakensis*, 1 *A. caviae*, 1 *A. media* and 1 *A. sobria*. 

**Figure 6:**
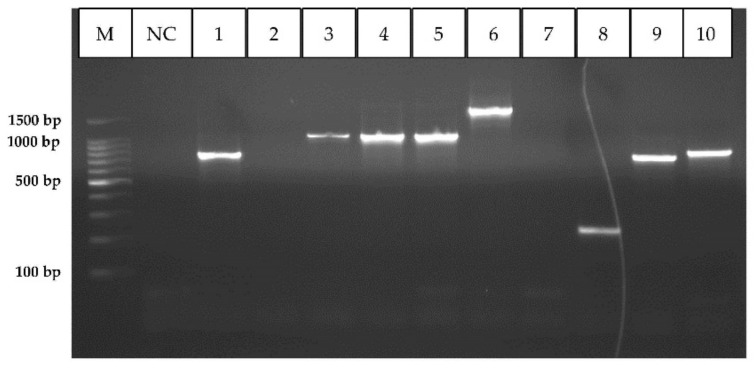
PCR gel image showing the positive amplicons of class 1 integron gene cassette regions. M: Molecular weight marker (100 bp), NC: negative control, Lane numbers 1,3–6,8–10—Positive; 2 and 7—Negative.

All isolates which showed the presence of either class 1 or class 2 integrons (*n* = 36), were amplified with class 1 and 2 integron gene cassette primers. Out of 35 class 1 integron positive aeromonads, 34 (94%) carried class 1 integron gene cassettes, which were ranging from approx. 250 bp–2000 bp (Figure 6). Twelve isolates carried class 2 integron gene cassette regions with fragment sizes ranging from 450 bp–1800 bp. A total of ten isolates contained both integron 1 and 2 gene cassettes (Supplementary Table S2).

A statistically significant (*p* < 0.01) association with presence of an integron (chi square 10.272, df 2, *p* = 0 0.005881) and presence of an integron 1 (chi square—9.3253, df 2, *p* = 0.009442) in the environmental and commensal *Aeromonas* isolates was found for scale of the farm. Results showed that there is statistical evidence that presence of an integron and integron 1 are significantly lower in small scale (*p* < 0.05) and medium scale (*p* < 0.01) farms compared to that of the reference group (large scale farms).

## 4. Discussion

Members of the genus *Aeromonas* are considered autochthonous in aquatic environments [1,45]. They have been frequently isolated from fresh water ornamental fish farming environments including in water, in sediment [46,47,48] and in apparently healthy and diseased fish [1,7,44]. The wide distribution of aeromonads in ornamental fish farming environment highlights their ability to serve as opportunistic pathogens in fish and also indicates their possible interactions with humans via direct contact or environmental contamination.

In our study, members of the genus *Aeromonas* comprised the majority among Gram-negative bacterial isolates (data not shown) from fish and their associated environment, in agreement with previous observations [7,49].

Identification of aeromonads based on morphological and biochemical characterization alone is often controversial and unreliable leading to erroneous identification [50]. In the present study, in agreement with previous findings, we encountered limitations in assigning bacterial isolates as members of the genus *Aeromonas* through isolation on selective media followed by phenotypic and biochemical characterization, as certain phenotypically identified aeromonads were later found to belong to other genera through generic characterization [1].

For the species identification of *Aeromonas* our study used sequencing of the *gyrB* gene that encodes the subunit B of DNA gyrase which was the first housekeeping gene studied for phylogenetic analysis of aeromonads [34]. Partial sequencing of the *gyrB* gene alone [6,19] or in combination with one or several other housekeeping genes [51,52] has been used successfully in many studies to characterize *Aeromonas* isolates in recent years. It is documented that the *gyrB* gene has a higher discriminatory power to differentiate between species and therefore is a suitable target for *Aeromonas* speciation [34]. It is noteworthy to mention that among 185 isolates which were biochemically confirmed as presumptive aeromonads and identified by *Aeromonas* genus specific 16S rDNA PCR, only 87% (161 isolates) was identified as members of *Aeromonas* by *gyrB* sequencing.

*Aeromonas* isolates recovered in this study (*n* = 161) belonged to eight different species confirming the considerably high diversity of aeromonads in ornamental fish and their environment, in agreement with previous studies [7,21,52]. Among our isolates, the overall prevalence of *A. veronii* was predominant (75.8%) while the prevalence of all other species was ≤5% except *A. hydrophila* (9.3%). Other comparable investigations of motile aeromonads from fresh water ornamental fish also have identified *A. veronii* as the most abundant species. In a study done by Dias et al. in 2012, among *Aeromonas* spp. isolated from water and skin of imported ornamental fish, *A. veronii* and *A*. *hydrophila* were identified as the most abundant species. Among 53 *Aeromonas* spp. isolated from septicaemic freshwater ornamental fish in Sri Lanka *A. veronii* represented 79.2% [53]. In a study done by Hossain et al. [6] *A. veronii* was the predominant species (65.4%) among aeromonads recovered from healthy guppy (*Poecilia reticulata*). Our findings were also comparable with a study done in China by Hu et al. [54] that reported *A. veronii* was the most common species isolated from healthy food fish (90⁄120; 75%) and water samples (25⁄40; 62.5%) while *A. veronii* (25⁄42; 60%) and *A. hydrophila* (14⁄42; 33%) was the species most commonly isolated from diseased fish.

However, according to a recent study [55] *A. sobria* (37%) and *A. hydrophila* (18%) were the species most frequently isolated from the kidney samples of 134 ornamental fish imported into Italy. Interestingly, 12% of the analyzed fish in their study were from Sri Lanka.

*A. hydrophila* represented only 9.3% of our isolates in disagreement with some of the earlier findings that reported *A. hydrophila* was the most abundant isolate with prevalence of 35.3% [56], 77% [1] and 50.65% [52].

*Aeromonas* spp. most frequently implicated in human infections, such as *A. hydrophila, A. caviae, A. veronii* and *A. dhakensis* [3,57] and their isolation from the ornamental fish culture environment and healthy fish in this study indicates that these sources serve as reservoirs of these pathogens. *A. hydrophila* and *A. veronii* are also the major causative agents of motile *Aeromonas* septicemia in ornamental fish [3] and their occurrence in fish farm environments may explain their opportunistic role as fish pathogens. Diseases in ornamental fish is the ultimate result of the disturbance of fine balance between the fish host, environment and pathogens. Ornamental fish are exposed to a wide range of stressors under intensive culture conditions that put them at a compromised state. Continuous and unavoidable exposure of such ornamental fish to these pathogens predispose them to opportunistic infections [58].

*Aeromonas* is a water-borne organism that is ubiquitous in aquatic environments. They readily acquire and exchange antimicrobial resistance genes (ARGs) [59] and are reported to be potential reservoirs of ARGs [21]. Due to this reason many authors have used *Aeromonas* as effective indicator organisms for monitoring AMR in aquatic environments [48]. Antimicrobial resistance in *Aeromonas* spp. from aquaculture is not a novel observation. Bacterial infections are common in intensively cultured aquatic animals, in particular in tropical ornamental fish, necessitating treatment with antimicrobial agents. As a result, the freshwater ornamental fish industry consumes antimicrobials and other chemicals in substantial quantities. Antimicrobials are added both therapeutically and prophylactically [16] to the feed or administered via water as a bath [13,14]. A fraction of the administered antimicrobials is passed unmetabolized through feces without complete decomposition [14] these persist and accumulate in the fish farming environment in sufficiently high concentrations to exert selective pressure on aquatic bacteria. This process drives the development of drug resistance in the aquatic microbiome. Hence, aquaculture systems are considered as “genetic reactors” or “hotspots” for the emergence of AMR [60].

When a microorganism shows resistance to at least one antimicrobial agent from each of three or more antimicrobial classes, that is defined as MDR [61]. *Aeromonas* with MDR strains have frequently been detected in many ornamental fish farming nations such as Thailand [7] as well as in imported countries including Portugal, South Korea and Italy [21,55,62]. This trend is concomitant with the industry expansion. Our results are consistent with previous studies, showing MDR *Aeromonas* strains are spreading in ornamental fish as well as in their associated environment.

In the present study, resistance of aeromonads to penicillins was widespread and 92.5% of the isolates showed resistance to amoxycillin, a beta-lactam antimicrobial. A high resistance to amoxycillin is attributed to the natural ability of aeromonads for β lactamase production and inducible β lactamase activity [3,44]. Our findings are consistent with those of previous studies which demonstrated that *Aeromonas* spp. are naturally resistant to penicillins [6,20,23,63]. Penicillin is one of the most commonly prescribed drug classes in humans and animals with numerous clinical indications and therefore, penicillin resistance is of clinical concern.

The very high level of resistance of *Aeromonas* spp. to quinolones observed in this study is worrying because quinolones are the first-line drugs recommended against infections caused by *Aeromonas* spp. Among our isolates, 67.1% were resistant to enrofloxacin which is a synthetic quinolone and 63.4% were resistant to nalidixic acid, a fluoroquinolone. In agreement with our finding high levels of resistance of aeromonads to nalidixic acid has been reported [23,62,64].

Tetracycline has been the commonest antimicrobial agent used in ornamental fish aquaculture for decades and therefore the observed level of resistance to tetracycline (23.6%) was not surprising. It is interesting to note that 37.3% of our isolates showed intermediate resistance to tetracycline. However, tetracycline resistance observed in this study was considerably lower than the levels reported in comparable studies in the recent past. Resistance levels as high as 79.69% [7] and 100% [6] have been detected in ornamental fish-borne aeromonads to oxytetracycline in Thailand and Korea, respectively. Acquired resistance to tetracycline has frequently been reported in aeromonads in aquaculture.

Level of resistance to erythromycin observed in our study was lower (26.1%) than the levels reported in comparable studies in other countries such as Portugal, Thailand, and Italy [7,21,55], as well as in Sri Lanka (54.7% of clinical isolates) [53] in aeromonads from ornamental fish.

Very low levels of resistance have been observed against chloramphenicol (3.7%) and nitrofurantoin (8.1%), two antimicrobials that are banned to use in livestock and aquaculture worldwide, which could probably be due to the misuse of these antimicrobials [64].

Many previous studies have reported 100% susceptibility of *Aeromonas* to the third generation cephalosporins [7,65]. However, 1.2% of our isolates were resistant to ceftazidime. Resistance to third-generation cephalosporin and imipenem is known to be associated with the depression of the chromosomal enzymes. It should be noted that the occurrence of resistance to imipenem, an antimicrobial belonging to the carbapenem group, was observed in 18% of isolates that belonged to *A. veronii, A. hydrophila* and *A. jandaei*. This is alarming given that this antimicrobial is not used (also not permitted to use) in Sri Lankan aquaculture. Moreover, Carbapenems are often used as “antibiotics of last resort” for the treatment of hospital-acquired infections caused by multidrug-resistant bacteria [66,67]. Human infections caused by Carbapenem resistant *Aeromonas* spp. have been reported in the recent past in Colombia and in United States [68,69]. Carbapenem resistance has been associated with the production of carbapenems hydrolyzing *Aeromonas* (CphA).

The high prevalence of multidrug resistant aeromonads in ornamental fish and their associated environment observed in this study is quite disturbing as these bacteria may pose a risk to the health and welfare of cultured fish as well as humans interacting with ornamental fish directly and indirectly such as farmers and hobbyists [9,70]. Infections caused by these resistant aeromonads can be difficult to treat due to both intrinsic and acquired AMR mechanisms.

The MAR index is calculated as the ratio between the number of antibiotics that an isolate is resistant to and the total number of antibiotics the organism is exposed to. It is considered a useful tool for risk assessment by identifying antimicrobial contamination from high-risk environment [71,72]. In our study, 110 isolates (68.3%) showed a MAR index value higher than 0.2 suggesting they originate from a high-risk source of contamination where antimicrobials are often used. Among those, 83 isolates (51.6%) showed MDR. The MAR index range in the current study (0.08–0.86) is comparatively wider when compared with previous studies. Jacobs and Chenia [44], John and Hatha [63], Igbinosa et al. [73], Hossain et al. [16] and Hossain et al. [6] have reported MAR indices ranging from 0.12–0.59, 0.08–0.5, 0.3–0.7, 0.22–0.56, and 0.28–0.67, respectively, in aquaculture-borne aeromonads. This indicates potential contamination with antimicrobials of aquatic environments in the sampled locations. Long term subtherapeutic concentrations of antimicrobials in aquatic environment could be a factor driving this process.

Integrons play a crucial role in multidrug resistance by acquisition of resistance gene cassettes [26]. They are often embedded in promiscuous plasmids and transposons, facilitating their lateral transfer into a wide range of pathogens [74]. According to Hall [27], the most common integron class present in Gram-negative bacteria is class 1. In the current study 21.7% of the isolates (*n* = 35) carried integrase 1 gene (class 1 integrons). This was lower than the levels reported in comparable studies. Higher incidence of class 1 integrons, such as 31% [1], 28.3% [64], and 64.62% [6] have been reported from ornamental fish borne bacteria. However, Otero-olarra and Curiel-quesada [52] has observed moderate incidence (24%) of class 1 integrons in accordance with our observations. Out of these 35 integrase 1 positive isolates, 34 (97.1%) contained gene cassettes with sizes ranging from 250 bp–approximately 2000 bp. This is a very high prevalence of gene cassettes compared to earlier reports of 35.71% [62] and 52.63% [6] from aquaculture-borne *Aeromonas* isolates. According to Ndi and Barton [1], a considerable number of integron positive isolates (5/28) were empty (150–250 bp) with no gene cassettes inserted between the conserved segments of the integron. Product sizes of integron class 1 gene cassette regions in the study was in agreement with Jacobs and Chenia [44] where they have observed products ranging from 300 bp–1500 bp.

Reports on the presence of Class 2 integrons in *Aeromonas* are very scarce. It is interesting to note that 7.45% (12/161) of our isolates carried class 2 integron gene cassette regions with fragment sizes ranging from 450 bp–1800 bp. The high incidence of Class 2 integrons was an important finding in this study as the presence of integron 2 has not been detected in similar studies done using aeromonads from ornamental fish and other freshwater fish [75]. Moreover, 6.8% of our strains contained both types of integrons. Presence of multi-drug resistant aeromonads bearing integrons in aquatic environment heightens the danger of co-selection and persistence of resistance determinants under the selective pressure imposed by the use of antimicrobial agents [75].

Our findings showed that the presence of multidrug resistance, an integron and an integron 1 were significantly lower in aeromonads in small scale farms compared to that of large-scale farms which may be, at least in part, due to the wider use of antimicrobials for disease control in large scale farms.

## 5. Conclusions

*A. veronii* found to be the predominant species among mesophilic, motile *Aeromonas* spp. isolated from healthy and diseased freshwater ornamental fish and their associated environment. The usefulness of *gyrB* sequencing in discrimination of *Aeromonas* species was confirmed. *Aeromonas* spp. was used as bacterial indicators of antimicrobial resistance. Our findings suggest that ornamental fish and farm effluent water act as a reservoir for multidrug resistant *Aeromonas* spp. bearing integrons. To our knowledge, this is the first report of the detection of class 1 and 2 integrons in motile *Aeromonas* species from healthy ornamental fish and their associated environment in Sri Lanka. The observed high level of imipenem resistance was also alarming. Our study provides baseline data on the levels of drug resistance in aeromonads from ornamental fish which will be essential to manage and mitigate potential risks to human and fish health and to safeguard the blooming ornamental fish industry in Sri Lanka.

In the present study, samples were collected from 24 ornamental fish farms where there was a wide diversity in the number of fish tanks per farm, type of fish tanks per farm (e.g., cement, glass etc.), number of fish per tank and number of different species per tank. This is in part due to the unregulated nature of this industry. Therefore, a wider and representative sampling method was not adopted considering the practicability of sampling. Moreover, due to the abundance of aeromonads in freshwater environments and fish, the present study included a limited number of *Aeromonas* isolates from each sampling source in each farm considering the feasibility of laboratory confirmation and costs involved. We emphasize that having a larger isolate collection representing all sampling sources would offer further insights into the levels of drug resistance and therefore should be considered in future research.

The rapid expansion and diversification of the ornamental aquaculture industry in recent decades has resulted in a concomitant increase in the use of drugs to combat infectious diseases. Antimicrobials present at subtherapeutic levels for prolonged periods in the water and sediments of ornamental fish farms provide ideal conditions for the emergence and selection of resistant bacterial strains. Education of all stakeholders about the detrimental effects of overuse/abuse of antimicrobials in fish, human beings and in aquatic ecosystems, encouragement in the use of environmentally friendly disease prevention measures and adoption of good husbandry practices must be the focus to minimize the use of antimicrobials in ornamental fish aquaculture.

## Figures and Tables

**Table 2:** Different sources of isolation of *Aeromonas* spp.

			*A. veronii*	*A. caviae*	*A. sobria*	*A. dhakensis*	*A. hydrophila*	*A. jandaei*	*A. media*	*A. popoffii*
Source										
	Aquatic environment									
		Effluent water	23			1	1			1
		Pond sediment	36	3	1					
		Biofilm	16	2		1		1		
	Apparently healthy ornamental fish									
		*Poecilia reticulata* (Guppy)	6			2	2			
		*Carassius auratus* (Goldfish)	11	1				1		
		*Xiphophorus helleri* (Swordtail)	4						1	
		*Xiphophorus maculatus* (Platy)	8	1						
		*Poecilia sphenops* (Molly)	2	1						
	Diseased fish		16			2	12	5		

**Table 3:** Distribution of antimicrobial susceptibility among *Aeromonas* spp. to thirteen antimicrobials.

		Amoxicillin	Nalidixic acid	Rifampicin	Chloramphenicol	Doxycycline	Erythromycin	Enrofloxacin	Tetracycline	Nitrofurantoin	Trimethoprim-sulfamethoxazole	Gentamicin	Ceftazidime	Imipenem
		n (%)	n (%)	n (%)	n (%)	n (%)	n (%)	n (%)	n (%)	n (%)	n (%)	n (%)	n (%)	n (%)
***A. veronii***	*R*	111 (91.0)	80 (65.6)	1 (0.8)	4 (3.3)	5 (4.1)	26 (21.3)	78 (63.9)	28 (23.0)	10 (8.2)	18 (14.8)	21 (17.2)	2 (1.6)	26 (21.3)
	*I*		2 (1.6)	5 (4.1)	3 (2.5)	17 (13.9)			52 (42.6)				3 (2.5)	40 (32.8)
	*S*	11 (9.0)	40 (32.8)	116 (95.1)	115 (94.3)	100 (82.0)	96 (78.7)	44 (36.1)	42 (34.4)	112 (91.8)	104 (85.2)	101 (82.8)	117 (95.9)	56 (45.9)
***A. caviae***	*R*	8 (100)	4 (50.0)				3 (37.5)	6 (75)	1 (12.5)					
	*I*					2 (25)			1 (12.5)					
	*S*		4 (50.0)	8 (100)	8 (100)	6 (75)	5 (62.5)	2 (25)	6 (75)	8 (100)	8 (100)	8 (100)	8 (100)	8 (100)
***A. sobria***	*R*	1 (100)	1 (100)					1 (100)	1 (100)					
	*I*					1 (100)								1 (100)
	*S*			1 (100)	1 (100)		1 (100)			1 (100)	1 (100)	1 (100)	1 (100)	
***A. dhakensis***	*R*	6 (100)	4 (66.7)	2 (33.3)	1 (16.7)	1 (16.7)	2 (33.3)	6 (100)	2 (33.3)	2 (33.3)	2 (33.3)			
	*I*								1 (16.7)			1 (16.7)	1 (16.7)	1 (16.7)
	*S*		2 (33.3)	4 (66.7)	5 (83.3)	5 (83.3)	4 (66.7)		3 (50.0)	4 (66.7)	4 (66.7)	5 (83.3)	5 (83.3)	5 (83.3)
***A. hydrophila***	*R*	15 (100)	7 (46.7)	1 (6.7)		2 (13.3)	8 (53.3)	11 (73.3)	5 (33.3)	1 (6.7)	3 (20.0)	2 (13.3)		2 (13.3)
	*I*			1 (6.7)		3 (20.0)		2 (13.3)	5 (33.3)					2 (13.3)
	*S*		8 (53.3)	13 (86.6)	15 (100)	10 (66.7)	7 (46.7)	2 (13.3)	5 (33.3)	14 (93.3)	12 (60.0)	13 (86.6)	15 (100)	11 (73.3)
***A. jandaei***	*R*	6 (85.7)	5 (71.4)		1 (14.3)		2 (28.6)	5 (71.4)	1 (14.3)		3 (42.9)	1 (14.3)		1 (14.3)
	*I*				1 (14.3)			1 (14.3)	1 (14.3)					6 (85.7)
	*S*	1 (14.3)	2 (28.6)	7 (100)	5 (71.4)	7 (100)	5 (71.4)	1 (14.3)	5 (71.4)	7 (100)	4 (57.1)	6 (85.7)	7 (100)	
***A. media***	*R*	1 (100)	1 (100)				1 (100)	1 (100)						
	*I*								1 (100)					1 (100)
	*S*			1 (100)	1 (100)	1 (100)				1 (100)	1 (100)	1 (100)	1 (100)	
***A. popoffii***	*R*	1 (100)												
	*I*													
	*S*		1 (100)	1 (100)	1 (100)	1 (100)	1 (100)	1 (100)	1 (100)	1 (100)	1 (100)	1 (100)	1 (100)	1 (100)

## Data Availability

The partial *gyrB* gene sequences of the isolates were deposited in GenBank, accession numbers LC644207 to LC644367.

## Supplementary Materials

The following are available online at https://www.mdpi.com/article/10.3390/microorganisms9102106/s1, Figure S1: Phylogenetic tree of *Aeromonas* spp. based on the *gyrB* gene sequences using neighbor-joining method with bootstrap replication of 1000, Table S1: Origin and species distribution of *Aeromonas* isolates, Table S2: Characterization of integrons and integron gene cassettes (class 1 and 2) in integron carrying *Aeromonas* isolates (*n* = 36).
