# Supplementary material for: Characterization and Antimicrobial Resistance of Environmental and Clinical Aeromonas Species Isolated from Fresh Water Ornamental Fish and Associated Farming Environment in Sri Lanka
Source: Microorganisms. 2021 Oct 6;9(10):2106. doi: 10.3390/microorganisms9102106 (PMC8537582; doi:10.3390/microorganisms9102106)
Supplement: Supplementary file 1 [file microorganisms-09-02106-s001.zip › Supplementary materials/Table S1_Origin and species distribution of Aeromonas isolates.pdf]

Table S1. Origin and species distribution ~~diversity~~ of *Aeromonas* isolates

	**Isolate**	**Source**	**Species**
**1**	AE1	Effluent water	*Aeromonas veronii*
**2**	AE2	Pond sediment	*Aeromonas veronii*
**3**	AH3	Skin mucous of apparently healthy guppy	*Aeromonas hydrophila*
**4**	AH4	Skin mucous of apparently healthy swordtail fish	*Aeromonas veronii*
**5**	AE5	Biofilm	*Aeromonas veronii*
**6**	AE6	Biofilm	*Aeromonas veronii*
**7**	AE7	Effluent water	*Aeromonas veronii*
**8**	AE8	Pond sediment	*Aeromonas veronii*
**9**	AH9	Skin mucous of apparently healthy guppy	*Aeromonas veronii*
**10**	AH10	Skin mucous of apparently healthy goldfish	*Aeromonas veronii*
**11**	AE12	Effluent water	*Aeromonas veronii*
**12**	AH15	Skin mucous of apparently healthy goldfish	*Aeromonas veronii*
**13**	AE19	Pond sediment	*Aeromonas veronii*
**14**	AH20	Skin mucous of apparently healthy guppy	*Aeromonas veronii*
**15**	AH21	Skin mucous of apparently healthy guppy	*Aeromonas hydrophila*
**16**	AH22	Skin mucous of apparently healthy goldfish	*Aeromonas veronii*
**17**	AH23	Skin mucous of apparently healthy goldfish	*Aeromonas veronii*
**18**	AE26	Effluent water	*Aeromonas veronii*
**19**	AE28	Effluent water	*Aeromonas veronii*
**20**	AE29	Pond sediment	*Aeromonas veronii*
**21**	AH31	Skin mucous of apparently healthy guppy	*Aeromonas veronii*
**22**	AE32	Effluent water	*Aeromonas veronii*
**23**	AE33	Pond sediment	*Aeromonas veronii*
**24**	AE34	Pond sediment	*Aeromonas veronii*
**25**	AE35	Pond sediment	*Aeromonas veronii*
**26**	AH37	Skin mucous of apparently healthy guppy	*Aeromonas media*
**27**	AH38	Skin mucous of apparently healthy guppy	*Aeromonas veronii*
**28**	AE39	Pond sediment	*Aeromonas veronii*
**29**	AE40	Pond sediment	*Aeromonas veronii*
**30**	AE41	Pond sediment	*Aeromonas caviae*
**31**	AE42	Biofilm	*Aeromonas veronii*
**32**	AE43	Biofilm	*Aeromonas veronii*
**33**	AH44	Skin mucous of apparently healthy guppy	*Aeromonas veronii*
**34**	AE46	Pond sediment	*Aeromonas veronii*
**35**	AH49	Skin mucous of apparently healthy guppy	*Aeromonas veronii*
**36**	AH50	Skin mucous of apparently healthy guppy	*Aeromonas veronii*
**37**	AE52	Effluent water	*Aeromonas veronii*
**38**	AE53	Pond sediment	*Aeromonas veronii*
**39**	AE54	Pond sediment	*Aeromonas veronii*
**40**	AE56	Biofilm	*Aeromonas caviae*
**41**	AH57	Skin mucous of apparently healthy molly	*Aeromonas veronii*
**42**	AH58	Skin mucous of apparently healthy molly	*Aeromonas veronii*
**43**	AH59	Skin mucous of apparently healthy platy fish	*Aeromonas caviae*
**44**	AE60	Effluent water	*Aeromonas veronii*
**45**	AE61	Pond sediment	*Aeromonas veronii*
**46**	AE62	Pond sediment	*Aeromonas veronii*
**47**	AH63	Skin mucous of apparently healthy platy fish	*Aeromonas veronii*
**48**	AE65	Biofilm	*Aeromonas veronii*

**49**	AE66	Effluent water	*Aeromonas veronii*
**50**	AE67	Effluent water	*Aeromonas veronii*
**51**	AE68	Pond sediment	*Aeromonas veronii*
**52**	AE70	Pond sediment	*Aeromonas veronii*
**53**	AH71	Skin mucous of apparently healthy platy fish	*Aeromonas veronii*
**54**	AH72	Skin mucous of apparently healthy swordtail fish	*Aeromonas veronii*
**55**	AE73	Biofilm	*Aeromonas veronii*
**56**	AE74	Effluent water	*Aeromonas veronii*
**57**	AE75	Pond sediment	*Aeromonas veronii*
**58**	AE76	Pond sediment	*Aeromonas veronii*
**59**	AE77	Biofilm	*Aeromonas veronii*
**60**	AE79	Effluent water	*Aeromonas veronii*
**61**	AE80	Pond sediment	*Aeromonas veronii*
**62**	AE81	Pond sediment	*Aeromonas veronii*
**63**	AE82	Pond sediment	*Aeromonas veronii*
**64**	AH83	Skin mucous of apparently healthy platy fish	*Aeromonas veronii*
**65**	AH84	Skin mucous of apparently healthy platy fish	*Aeromonas veronii*
**66**	AE85	Biofilm	*Aeromonas veronii*
**67**	AE86	Biofilm	*Aeromonas dhakensis*
**68**	AE87	Effluent water	*Aeromonas dhakensis*
**69**	AE88	Pond sediment	*Aeromonas veronii*
**70**	AH90	Skin mucous of apparently healthy guppy	*Aeromonas veronii*
**71**	AH91	Skin mucous of apparently healthy goldfish	*Aeromonas veronii*
**72**	AE92	Biofilm	*Aeromonas veronii*
**73**	AE93	Effluent water	*Aeromonas veronii*
**74**	AE94	Pond sediment	*Aeromonas veronii*
**75**	AE95	Pond sediment	*Aeromonas caviae*
**76**	AH96	Skin mucous of apparently healthy goldfish	*Aeromonas veronii*
**77**	AE97	Effluent water	*Aeromonas veronii*
**78**	AE98	Effluent water	*Aeromonas veronii*
**79**	AE99	Pond sediment	*Aeromonas veronii*
**80**	AH100	Skin mucous of apparently healthy guppy	*Aeromonas veronii*
**81**	AH101	Skin mucous of apparently healthy goldfish	*Aeromonas veronii*
**82**	AE102	Biofilm	*Aeromonas veronii*
**83**	AE103	Effluent water	*Aeromonas veronii*
**84**	AE105	Pond sediment	*Aeromonas caviae*
**85**	AH106	Skin mucous of apparently healthy goldfish	*Aeromonas veronii*
**86**	AH107	Skin mucous of apparently healthy platy fish	*Aeromonas veronii*
**87**	AE108	Biofilm	*Aeromonas veronii*
**88**	AE111	Pond sediment	*Aeromonas veronii*
**89**	AH112	Skin mucous of apparently healthy goldfish	*Aeromonas caviae*
**90**	AH113	Skin mucous of apparently healthy molly	*Aeromonas caviae*
**91**	AE115	Biofilm	*Aeromonas veronii*
**92**	AE117	Effluent water	*Aeromonas veronii*
**93**	AE118	Effluent water	*Aeromonas veronii*
**94**	AE119	Pond sediment	*Aeromonas veronii*
**95**	AE120	Pond sediment	*Aeromonas veronii*
**96**	AH122	Skin mucous of apparently healthy guppy	*Aeromonas veronii*
**97**	AE123	Biofilm	*Aeromonas veronii*
**98**	AE125	Effluent water	*Aeromonas veronii*
**99**	AE127	Pond sediment	*Aeromonas veronii*

**100**	AE130	Biofilm	*Aeromonas veronii*
**101**	AE131	Effluent water	*Aeromonas popoffii*
**102**	AE132	Pond sediment	*Aeromonas veronii*
**103**	AE133	Pond sediment	*Aeromonas veronii*
**104**	AH134	Skin mucous of apparently healthy platy fish	*Aeromonas veronii*
**105**	AH135	Skin mucous of apparently healthy guppy	*Aeromonas dhakensis*
**106**	AH136	Skin mucous of apparently healthy guppy	*Aeromonas dhakensis*
**107**	AE138	Effluent water	*Aeromonas veronii*
**108**	AE141	Pond sediment	*Aeromonas veronii*
**109**	AE142	Pond sediment	*Aeromonas veronii*
**110**	AE144	Pond sediment	*Aeromonas veronii*
**111**	AH147	Skin mucous of apparently healthy guppy	*Aeromonas jandaei*
**112**	AH149	Skin mucous of apparently healthy guppy	*Aeromonas veronii*
**113**	AE150	Biofilm	*Aeromonas caviae*
**114**	AE151	Effluent water	*Aeromonas veronii*
**115**	AE153	Pond sediment	*Aeromonas veronii*
**116**	AE154	Pond sediment	*Aeromonas veronii*
**117**	AE155	Pond sediment	*Aeromonas sobria*
**118**	AH156	Skin mucous of apparently healthy guppy	*Aeromonas veronii*
**119**	AE157	Biofilm	*Aeromonas veronii*
**120**	AE159	Effluent water	*Aeromonas veronii*
**121**	AE160	Effluent water	*Aeromonas veronii*
**122**	AE163	Pond sediment	*Aeromonas veronii*
**123**	AH164	Skin mucous of apparently healthy guppy	*Aeromonas veronii*
**124**	AE165	Biofilm	*Aeromonas jandaei*
**125**	AE167	Biofilm	*Aeromonas veronii*
**126**	AC1	Wound swab of giant gourami	*Aeromonas dhakensis*
**127**	AC3	Wound swab of giant gourami	*Aeromonas hydrophila*
**128**	AC4	Wound swab of giant gourami	*Aeromonas hydrophila*
**129**	AC7	Kidney culture of guppy	*Aeromonas jandaei*
**130**	AC8	Kidney culture of guppy	*Aeromonas jandaei*
**131**	AC9	Wound swab of giant gourami	*Aeromonas hydrophila*
**132**	AC11	Wound swab of platy	*Aeromonas hydrophila*
**133**	AC12	Kidney culture of platy	*Aeromonas hydrophila*
**134**	AC14	Kidney culture of platy	*Aeromonas hydrophila*
**135**	AC15	Kidney culture of platy	*Aeromonas hydrophila*
**136**	AC16	Wound swab of giant gourami	*Aeromonas veronii*
**137**	AC17	Kidney culture of koi carp	*Aeromonas veronii*
**138**	AC18	Kidney culture of koi carp	*Aeromonas veronii*
**139**	AC19	Kidney culture of discus	*Aeromonas veronii*
**140**	AC21	Wound swab of silver dollar	*Aeromonas veronii*
**141**	AC22	Wound swab of silver dollar	*Aeromonas veronii*
**142**	AC23	Kidney culture of goldfish	*Aeromonas veronii*
**143**	AC25	Kidney culture of platy	*Aeromonas veronii*
**144**	AC26	Kidney culture of platy	*Aeromonas veronii*
**145**	AC27	Kidney culture of Oscar	*Aeromonas hydrophila*
**146**	AC29	Kidney culture of guppy	*Aeromonas veronii*
**147**	AC30	Kidney culture of guppy	*Aeromonas veronii*
**148**	AC31	Kidney culture of guppy	*Aeromonas veronii*
**149**	AC32	Kidney culture of guppy	*Aeromonas veronii*
**150**	AC34	Kidney culture of goldfish	*Aeromonas veronii*

**151**	AC35	Kidney culture of goldfish	*Aeromonas hydrophila*
**152**	AC36	Kidney culture of goldfish	*Aeromonas jandaei*
**153**	AC37	Kidney culture of goldfish	*Aeromonas veronii*
**154**	AC39	Wound swab of Oscar	*Aeromonas hydrophila*
**155**	AC40	Kidney culture of goldfish	*Aeromonas jandaei*
**156**	AC42	Kidney culture of guppy	*Aeromonas jandaei*
**157**	AC43	Wound swab of giant gourami	*Aeromonas hydrophila*
**158**	AC44	Wound swab of goldfish	*Aeromonas dhakensis*
**159**	AC45	Kidney culture of guppy	*Aeromonas veronii*
**160**	AC46	Wound swab of giant gourami	*Aeromonas hydrophila*
**161**	AE168	Effluent water	*Aeromonas hydrophila*
