# Supplementary material for: Characterization and Antimicrobial Resistance of Environmental and Clinical Aeromonas Species Isolated from Fresh Water Ornamental Fish and Associated Farming Environment in Sri Lanka
Source: Microorganisms. 2021 Oct 6;9(10):2106. doi: 10.3390/microorganisms9102106 (PMC8537582; doi:10.3390/microorganisms9102106)
Supplement: Supplementary file 1 [file microorganisms-09-02106-s001.zip › Supplementary materials/Table S2_Characterization of integrons and integron gene cassettes.pdf]

Table S2. Characterization of integrons and integron gene cassettes (class 1 and class 2) in integron carrying *Aeromonas* isolates (*n*=36)

Isolate	Species	Integron *Integrase* PCR	*Integrase 1* PCR	Amplicon size of class 1 integron gene cassette (bp)	Amplicon size of class 2 integron gene cassette (bp)
AH37	*Aeromonas media*	+	+	800	
AE41	*Aeromonas caviae*	-	+	250	1200
AE52	*Aeromonas veronii*	+	+	1200	
AH57	*Aeromonas veronii*	+	+	1200	
AH58	*Aeromonas veronii*	+	+	1200	800
AH63	*Aeromonas veronii*	+	+	1800	
AE81	*Aeromonas veronii*	-	+		800
AH83	*Aeromonas veronii*	+	+	250	
AH90	*Aeromonas veronii*	+	+	800	850
AH91	*Aeromonas veronii*	+	+	900	
AH96	*Aeromonas veronii*	+	-	1800	
AE118	*Aeromonas veronii*	+	+	900	
AE120	*Aeromonas veronii*	+	+	900	1800
AE125	*Aeromonas veronii*	+	+	800	600
AE132	*Aeromonas veronii*	+	+	1800	
AE133	*Aeromonas veronii*	+	+	1500	
AH135	*Aeromonas dhakensis*	+	+	1800	
AH136	*Aeromonas dhakensis*	+	+	2000	
AE141	*Aeromonas veronii*	-	+	1100	800
AE144	*Aeromonas veronii*	+	+	800	
AH147	*Aeromonas jandaei*	+	+	1350	1800
AE153	*Aeromonas veronii*	+	+	750	
AE154	*Aeromonas veronii*	+	+	750	
AE155	*Aeromonas sobria*	-	+	750	
AE157	*Aeromonas veronii*	+	+	750	
AH164	*Aeromonas veronii*	+	+	1800	
AE167	*Aeromonas veronii*	+	+	750	
AC1	*Aeromonas dhakensis*	+	-		1100
AC7	*Aeromonas. jandaei*	+	+	1800	
AC8	*Aeromonas jandaei*	+	+	1800	
AC11	*Aeromonas hydrophila*	+	+	1800	
AC12	*Aeromonas hydrophila*	+	+	1800	
AC16	*Aeromonas veronii*	+	+	1800	800
AC25	*Aeromonas veronii*	+	+	1100	
AC31	*Aeromonas veronii*	+	+	1500	450
AC42	*Aeromonas jandaei*	+	+	1300	1800
