# Supplementary figures and images for: Characterization and Antimicrobial Resistance of Environmental and Clinical Aeromonas Species Isolated from Fresh Water Ornamental Fish and Associated Farming Environment in Sri Lanka
Source: Microorganisms. 2021 Oct 6;9(10):2106. doi: 10.3390/microorganisms9102106 (PMC8537582; doi:10.3390/microorganisms9102106)

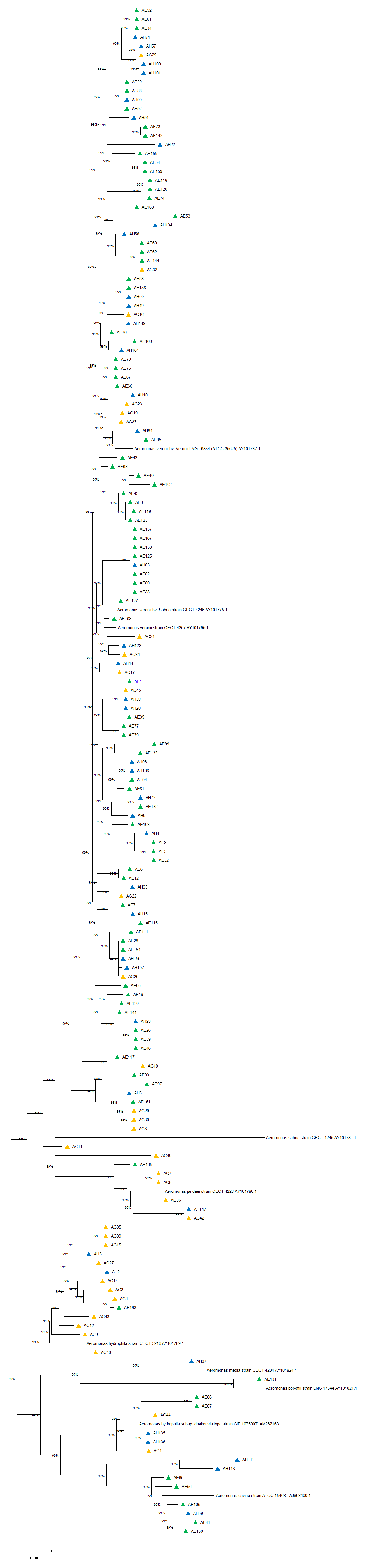

Supplement: Supplementary file 1 [file microorganisms-09-02106-s001.zip › Supplementary materials/Figure S1_Phylogenetic tree of Aeromonas spp. based on the gyrB gene sequences using neighbour-joining method with bootstrap replication of 1000.tif]
